# Novel Nargenicin A1 Analog Inhibits Angiogenesis by Downregulating the Endothelial VEGF/VEGFR2 Signaling and Tumoral HIF-1α/VEGF Pathway

**DOI:** 10.3390/biomedicines8080252

**Published:** 2020-07-29

**Authors:** Jang Mi Han, Ye Seul Choi, Dipesh Dhakal, Jae Kyung Sohng, Hye Jin Jung

**Affiliations:** 1 Department of Life Science and Biochemical Engineering, Sun Moon University, Asan 31460, Korea; gkswkdal200@naver.com (J.M.H.); yesll96@naver.com (Y.S.C.); medipesh@gmail.com (D.D.); sohng@sunmoon.ac.kr (J.K.S.); 2 Department of Pharmaceutical Engineering and Biotechnology, Sun Moon University, Asan 31460, Korea

**Keywords:** angiogenesis, nargenicin A1, compound **9**, VEGF, VEGFR2, HIF-1α

## Abstract

Targeting angiogenesis is an attractive strategy for the treatment of angiogenesis-related diseases, including cancer. We previously identified 23-demethyl 8,13-deoxynargenicin (compound **9**) as a novel nargenicin A1 analog with potential anticancer activity. In this study, we investigated the antiangiogenic activity and mode of action of compound **9**. This compound was found to effectively inhibit in vitro angiogenic characteristics, including the proliferation, invasion, capillary tube formation, and adhesion of human umbilical vein endothelial cells (HUVECs) stimulated by vascular endothelial growth factor (VEGF). Furthermore, compound **9** suppressed the neovascularization of the chorioallantoic membrane of growing chick embryos in vivo. Notably, the antiangiogenic properties of compound **9** were related to the downregulation of VEGF/VEGFR2-mediated downstream signaling pathways, as well as matrix metalloproteinase (MMP)-2 and MMP-9 expression in HUVECs. In addition, compound **9** was found to decrease the in vitro AGS gastric cancer cell-induced angiogenesis of HUVECs by blocking hypoxia-inducible factor-1α (HIF-1α) and VEGF expression in AGS cells. Collectively, our findings demonstrate for the first time that compound **9** is a promising antiangiogenic agent targeting both VEGF/VEGFR2 signaling in ECs and HIF-1α/VEGF pathway in tumor cells.

## 1. Introduction

Angiogenesis leads to the formation of new blood vessels by endothelial cells (ECs) from existing vessels. This process plays a crucial role in the growth, metastasis, and progression of solid tumors by providing oxygen, nutrients, and growth factors [1]. Therefore, targeting angiogenesis is regarded as a key strategy for the effective treatment of cancer [2,3]. Angiogenesis involves the enzymatic degradation of the vascular endothelial matrix and EC migration, adhesion, proliferation, and tube formation [4]. Diverse bioactive compounds with the antiangiogenic activities are involved in the downregulation of angiogenesis [5,6].

Tumor angiogenesis is generally initiated by various proangiogenic factors secreted from tumor cells. Vascular endothelial growth factor (VEGF) is the most potent inducer of angiogenesis and interacts with the tyrosine kinase VEGF receptors (VEGFRs), including VEGFR1, VEGFR2, and VEGFR3 [7]. Among these, VEGFR2-mediated signaling prominently induces cellular responses involved in angiogenesis. The binding of VEGF to VEGFR2 results in autophosphorylation of specific tyrosine residues in the cytoplasmic domain of VEGFR2 and sequentially promotes the activation of its downstream signaling effectors, including signal transducer and activator of transcription 3 (STAT3), serine/threonine protein kinase B (AKT), and extracellular signal-regulated kinase 1/2 (ERK1/2) [8,9,10,11]. Accordingly, blocking the VEGF/VEGFR2 signaling pathways is a powerful technique to disrupt tumor angiogenesis and tumor growth.

**Figure 1:**
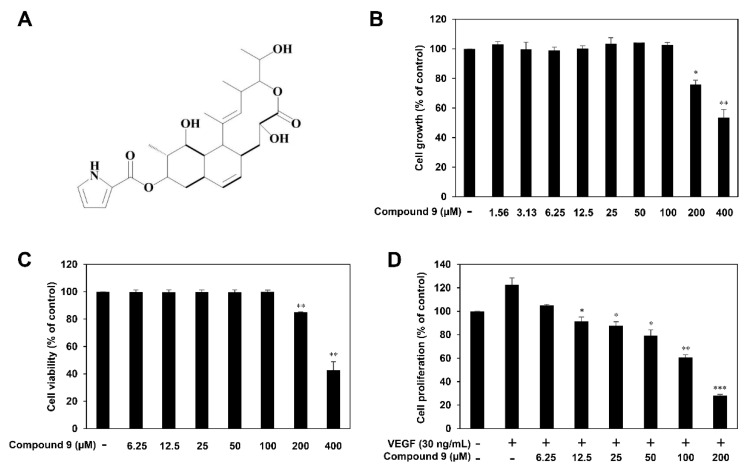
(**A**) The chemical structure of 23-demethyl 8,13-deoxynargenicin (compound **9**). (**B**,**C**) The cytotoxic effect of compound **9** on HUVECs. Cells were treated with the indicated concentrations of compound **9** for 24 h. (**B**) Cell growth was measured by the MTT assay and the IC_50_ value from obtained data was analyzed using the curve-fitting program GraphPad Prism 5. (**C**) Cell viability was measured by the trypan blue exclusion method. * *p* < 0.05, ** *p* < 0.01 vs. the control. (**D**) The antiproliferative effect of compound **9** in HUVECs. Cells were treated with the indicated concentrations (6.25–200 μM) of compound **9** in the presence of VEGF (30 ng/mL) for 72 h. * *p* < 0.05, ** *p* < 0.01, *** *p* < 0.001 vs. the VEGF control. Each value represents the mean ± SD from three independent experiments.

Nargenicin A1 is the major secondary metabolite produced by *Nocardia* species and a macrolide compound with effective antibacterial activity against various Gram-positive pathogenic bacteria [12]. In previous studies, several metabolic engineering and enzymatic modification approaches have been applied to boost production and generate novel glycosylated derivatives of nargenicin A1 [13,14]. More recently, we characterized the tailoring steps for the biosynthesis of nargenicin A1 in *Nocardia* sp. CS682, which resulted in the generation of several new analogs of the natural product [15]. An analysis of the bioactivity of the analogs revealed that 23-demethyl 8,13-deoxynargenicin (compound **9**) possesses potential antitumor activity, unlike nargenicin A1 and the other analogs (Figure 1A). Compound **9** suppressed the growth of various cancer cell lines, including gastric, lung, skin, liver, colon, brain, breast, and cervical cancer, within a range of concentrations that did not affect normal cell growth [15]. Further analysis of the underlying molecular mechanisms of its anticancer effect demonstrated that compound **9** exhibits a growth inhibitory effect against AGS gastric cancer cells by inducing G2/M cell cycle arrest, reactive oxygen species (ROS)- and caspase-mediated apoptosis, and autophagy by downregulating the phosphatidylinositol 3-kinase (PI3K)/AKT/mammalian target of rapamycin (mTOR) pathway [15]. Although we identified the anticancer activity of compound **9**, its effect on angiogenesis and VEGF/VEGFR2 signaling has not yet been studied.

In the present study, we assessed the antiangiogenic activity of compound **9** using human umbilical vein endothelial cells (HUVECs). Compound **9** inhibited VEGF-induced angiogenesis by suppressing the proliferation, invasion, tube formation, and adhesion of HUVECs. The antiangiogenic activity of compound **9** was verified using the chorioallantoic membrane (CAM) assay in vivo. Compound **9** was found to downregulate the VEGFR2-mediated signaling pathways, as well as matrix metalloproteinase (MMP) expression. Furthermore, compound **9** inhibited the AGS gastric cancer cell-induced angiogenesis of HUVECs by decreasing the expression of hypoxia-inducible factor-1α (HIF-1α) and VEGF. Therefore, we propose that compound **9** has the potential to regress tumor progression by targeting angiogenesis.

## 2. Experimental Section

### 2.1. Materials

Compound **9** was isolated from the culture extract of the constructed *Nocardia* sp. CS682 mutant, as shown in our previous report [15] and prepared at a concentration of 100 mM using dimethyl sulfoxide (DMSO). Endothelial growth medium-2 (EGM-2) and antibiotics were purchased from Lonza (Walkersville, MD, USA), and fetal bovine serum (FBS) and RPMI-1640 medium were purchased from Invitrogen (Grand Island, NY, USA). Recombinant human vascular endothelial growth factor 165 (VEGF165), Transwell chamber system, and Matrigel were purchased from Koma Biotech (Seoul, Korea, cat. no. K0921148), Corning Costar (Acton, MA, USA), and BD Biosciences (San Jose, CA, USA), respectively. Gelatin, trypan blue, and 3-[4,5-dimethylthiazol-2-yl]2,5-diphenyl tetrazolium bromide (MTT) were purchased from Sigma-Aldrich (St. Louis, MO, USA). Antibodies against VEGFR2 (cat. no. 2479), phospho-VEGFR2 (cat. no. 2478), STAT3 (cat. no. 9139), phospho-STAT3 (cat. no. 9131), AKT (cat. no. 9272), phospho-AKT (cat. no. 4060), ERK1/2 (cat. no. 9102), phospho-ERK1/2 (cat. no. 9101), MMP-2 (cat. no. 4022), MMP-9 (cat. no. 3852), HIF-1α (cat. no. 3716), and β-actin (cat. no. 4967) were purchased from Cell Signaling Technology (Danvers, MA, USA).

### 2.2. Cell Culture and Hypoxic Conditions

Human umbilical vein endothelial cells (HUVECs) and AGS human gastric cancer cells were obtained from American Type Culture Collection (Manassas, VA, USA) and Korean Cell Line Bank (Seoul, Korea), respectively. HUVECs and AGS cells were grown in EGM-2 and RPMI supplemented with 10% fetal bovine serum (FBS). The cells were maintained at 37 °C in a humidified 5% CO_2_ incubator (Thermo Scientific, Vantaa, Finland). For hypoxic conditions, the cells were incubated in a hypoxic chamber (SANYO, Chuou-ku, Osaka, Japan) under 5% CO_2_ and 1% O_2_ balanced with N_2_.

### 2.3. Cell Viability Assay

HUVECs (1 × 10^4^ cells/well) were seeded in 12-well culture plates and then treated with various concentrations of compound **9** (6.25–400 µM) for 24 h. The cells were stained with trypan blue and counted by a hemocytometer using an optical microscope (Olympus, Tokyo, Japan) at 200× magnification.

### 2.4. Cell Proliferation Assay

HUVECs (3 × 10^3^ cells/well) were seeded in 96-well culture plates and then treated with various concentrations of compound **9** (6.25–200 µM) in the presence of VEGF (30 ng/mL) for 72 h. Cell proliferation was measured using a 3-(4,5-dimethylthiazol-2-yl)-2,5-diphenyltetrazolium bromide (MTT) colorimetric assay.

### 2.5. Chemoinvasion Assay

The invasiveness of HUVECs was investigated using a Transwell chamber system with polycarbonate filter inserts with a pore size of 8.0 µm. The lower surface of the filter was coated with 10 µL of gelatin (1 mg/mL) for 1 h, and the upper surface was coated with 10 µL of Matrigel (3 mg/mL) for 1 h. Serum-starved HUVECs (8 × 10^4^ cells) were seeded in the upper chamber of the filter, and compound **9** (25–200 µM) were added to the lower chamber in the presence of VEGF (30 ng/mL). The chamber was incubated at 37 °C for 18 h, and then the cells were fixed with 70% methanol and stained with hematoxylin and eosin (H&E) at room temperature for 5 min. The total invaded cells were photographed and counted in randomly selected fields using an optical microscope (Olympus) at 200× magnification.

### 2.6. Capillary Tube Formation Assay

Serum-starved HUVECs (2 × 10^4^ cells) were placed on a surface containing Matrigel (10 mg/mL) using an angiogenesis kit (Ibidi GmbH, Munich, Germany) and incubated with compound **9** (25–200 µM) for 6 h in the presence of VEGF (30 ng/mL). The morphological changes and the tube formation of the cells were visualized under an optical microscope (Olympus) and photographed at 100× magnification. The number of tubes formed in the cells was counted in randomly selected fields at 100× magnification.

### 2.7. Adhesion Assay

The cell-matrix adhesion assay was performed in a 24-well culture plate coated with gelatin overnight at 4 °C. HUVECs (6 × 10^4^ cells/well) were seeded in each well and treated with compound **9** (25–200 µM) in the presence of VEGF (30 ng/mL). After 3 h, the unbound cells were carefully removed, and the attached cells were visualized and counted in randomly selected fields under a microscope at 200× magnification.

### 2.8. Chorioallantoic Membrane (CAM) Assay

Fertilized chick eggs were incubated in a humidified egg incubator at 37 °C and 50% humidity for 3 days. After incubation, approximately 6–9 mL of egg albumin was removed with a 10 mL hypodermic needle, allowing the CAM and yolk sac to drop away from the shell membrane. After 2 days, a small hole was punched on the broad end of the egg, and a window was carefully peeled away on the eggshell. Thermanox coverslips (Nalge Nunc International, Rochester, NY, USA) saturated with or without compound **9** (10 µg/egg) were air-dried and placed on the CAM surface. The windows were then sealed with cellophane tape. Two days later, 2 mL of 10% fat emulsion (Sigma-Aldrich) was injected into the chorioallantois and the vascular images were photographed.

### 2.9. Western Blot Analysis

After treatment, the cells were collected and lysed using RIPA buffer (Sigma-Aldrich) supplemented with a protease inhibitor cocktail (Roche Diagnostics, Indianapolis, IN, USA) on ice. Equal amounts of lysates were separated using 10% sodium dodecyl sulfate-polyacrylamide gel electrophoresis (SDS-PAGE). The separated proteins were then transferred to polyvinylidene difluoride (PVDF) membranes (EMD Millipore, Hayward, CA, USA) and blocked using Tris-buffered saline with Tween-20 (TBST) containing 5% skim milk at room temperature for 1 h. The membranes were then incubated with primary antibodies against phospho-VEGFR2 (dilution 1:2000), VEGFR2 (dilution 1:2000), phospho-STAT3 (dilution 1:2000), STAT3 (dilution 1:2000), phospho-AKT (dilution 1:2000), AKT (dilution 1:2000), phospho-ERK1/2 (dilution 1:2000), ERK1/2 (dilution 1:2000), MMP-2 (dilution 1:2000), MMP-9 (dilution 1:2000), HIF-1α (dilution 1:2000), and β-actin (dilution 1:2000) overnight at 4 °C. After washing with TBST three times, the membranes were incubated with horseradish peroxidase-conjugated anti-rabbit (dilution 1:3000) or anti-mouse (dilution 1:3000) secondary antibody for 1 h at room temperature. Immunolabeling was detected using an enhanced chemiluminescence (ECL) kit (Bio-Rad Laboratories, Hercules, CA, USA) according to the manufacturer’s instructions.

### 2.10. Tumor Cell-Induced Chemoinvasion Assay

A tumor cell-induced chemoinvasion assay was performed using an in vitro co-culture system based on the chemoinvasion assay. AGS cells (1 × 10^5^ cells/well) were seeded in the lower chamber and treated with compound **9** (50–200 μM) for 24 h. Then, the medium in each lower chamber was replaced with fresh medium without compound **9**, and serum-starved HUVECs (8 × 10^4^ cells) were placed in the upper chamber. The chamber was incubated at 37 °C for 18 h. The HUVECs that invaded the lower chamber of the filter were analyzed using the same procedure as in the chemoinvasion assay.

### 2.11. Tumor Cell-Induced Capillary Tube Formation Assay

To perform the tumor cell-induced capillary tube formation assay, conditioned medium was obtained from AGS cells and used as the angiogenic stimulus for tube formation in HUVECs. Briefly, AGS cells were treated with compound **9** (50–200 μM) for 24 h. The medium was then replaced with fresh medium without compound **9**.

### 2.12. Measurement of VEGF by Enzyme-Linked Immunosorbent Assay

The VEGF concentration in the AGS cells was measured using a VEGF immunoassay kit (R&D Systems, Minneapolis, MN, USA). Cells were incubated with or without compound **9** (50–200 μM) for 11 h under the indicated conditions. The supernatants were collected and the VEGF protein levels were measured according to the manufacturer’s instructions.

### 2.13. Statistical Analysis

The data are presented as the mean ± standard deviation (SD) of three independent experiments. Differences among groups were analyzed using analysis of variance (ANOVA) with SPSS statistics package (SPSS 9.0; SPSS Inc., Chicago, IL, USA). Post-hoc analysis was carried out using Tukey’s test. A *p*-value of <0.05 was considered to indicate a statistically significant difference.

## 3. Results

### 3.1. Effect of Compound ***9*** on In Vitro Angiogenesis of HUVECs

Prior to assessing the antiangiogenic properties of compound **9**, the cytotoxic effect of compound **9** on HUVECs was first investigated using the MTT assay and trypan blue exclusion method. Compound **9** inhibited the growth of HUVECs, with IC_50_ value of 412.3 μM (Figure 1B). Treatment with 6.25–100 μM of compound **9** did not affect the viability of HUVECs, whereas the cell viability was 85 and 43% after treatment with 200 and 400 μM of compound **9**, respectively (Figure 1C). Thus, the effects of compound **9** on in vitro angiogenesis were evaluated at concentrations up to 200 μM with low cytotoxicity.

**Figure 2:**
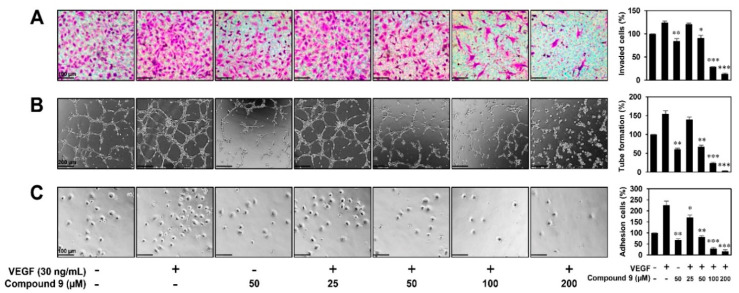
The in vitro antiangiogenic activity of compound **9** in HUVECs. (**A**–**C**) The inhibitory effects of compound **9** on the (**A**) invasion, (**B**) tube formation, and (**C**) adhesion of HUVECs induced by VEGF. Serum-starved HUVECs were stimulated with VEGF (30 ng/mL) in the presence or absence of compound **9** (25–200 μM). The basal levels of invasion, tube formation, and adhesion of HUVECs that were incubated in serum-free medium without VEGF were normalized to 100%. * *p* < 0.05, ** *p* < 0.01, *** *p* < 0.001 vs. the VEGF control. Each value represents the mean ± SD from three independent experiments.

We next investigated the effects of compound **9** on key steps in VEGF-induced angiogenesis, including EC proliferation, invasion, tube formation, and adhesion. As shown in Figure 1D, the MTT assay revealed that compound **9** dose-dependently inhibited the VEGF-stimulated proliferation of HUVECs. We also examined whether compound **9** affects the invasive migration of HUVECs using the Matrigel chemoinvasion assay. Treatment with 50–200 μM of compound **9** significantly suppressed the invasion of HUVECs stimulated by VEGF (Figure 2A). Furthermore, the Matrigel-based tube formation assay revealed that 50–200 μM of compound **9** markedly disrupted the tubular structures of HUVECs induced by VEGF (Figure 2B). Its effect on EC-matrix adhesion was assessed, and 25–200 μM of compound **9** was found to effectively reduce the VEGF-stimulated adhesion of HUVECs to gelatin (Figure 2C). When compared to compound **9** treatment alone in the absence of VEGF, 50 μM of compound **9** inhibited the VEGF-induced angiogenic phenotypes to the basal levels as shown in Figure 2A–C. In addition, cytotoxicity was not observed at 25–100 μM of concentrations by trypan blue staining performed in parallel to the in vitro angiogenesis assays. However, treatment with 200 μM of compound **9** exhibited the cytotoxic effect on HUVECs. These findings indicate that compound **9** inhibits VEGF-induced angiogenesis in the concentration range of 25–100 μM where cytotoxicity was not observed.

### 3.2. Effect of Compound ***9*** on In Vivo Angiogenesis

**Figure 3:**
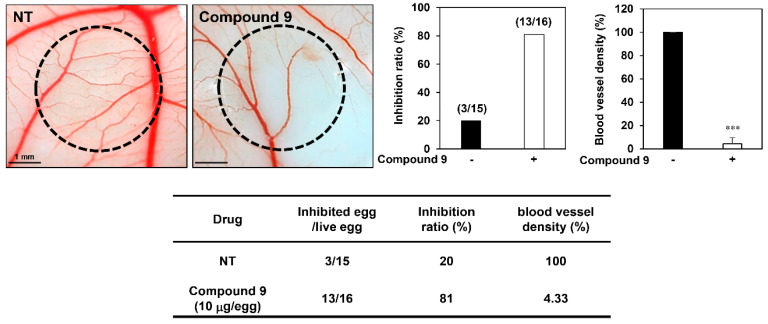
The in vivo antiangiogenic activity of compound **9** in CAMs. Fertilized chick eggs were maintained in a humidified egg incubator at 37 °C. At embryonic day 5, coverslips filled with vehicle alone or compound **9** (10 μg/egg) were placed to the CAM surface. Two days later, the vascular images were photographed. Calculations were based on the ratio of inhibited eggs relative to the total number of live eggs. Microvessel density was counted and the basal levels of control were normalized to 100%. *** *p* < 0.001 vs. the control.

We further validated the potential of compound **9** to suppress angiogenesis using the chick embryo CAM assay. As shown in Figure 3, compound **9** showed a significantly stronger inhibitory activity on CAM microvessel formation compared to the control group, with inhibition ratios of 81% and 20%, respectively. Furthermore, the blood vessel density was quantified and markedly decreased by compound **9** treatment without toxicity against pre-existing vessels. These results demonstrate that compound **9** exhibits promising antiangiogenic activity both in vitro and in vivo.

### 3.3. Effect of Compound ***9*** on VEGFR2-Mediated Downstream Signaling Pathways

**Figure 4:**
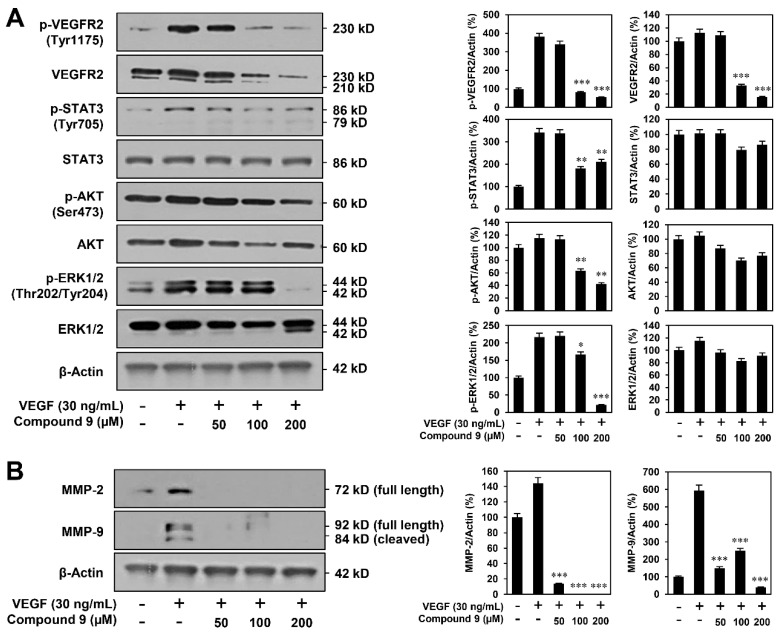
The downregulation of VEGF/VEGFR2-mediated signaling by compound **9**. (**A**) VEGFR2 downstream signaling inhibitory activities of compound **9**. Serum-starved HUVECs were pretreated with compound **9** (50–200 μM) for 1 h and then stimulated with VEGF (30 ng/mL) for 10 min. (**B**) MMP-2 and MMP-9 inhibitory activities of compound **9**. Serum-starved HUVECs were treated with compound **9** (50–200 μM) in the presence of VEGF (30 ng/mL) for 24 h. (**A**,**B**) Protein levels were detected by Western blot analysis and further quantified by densitometry. The level of β-actin was used as an internal control. * *p* < 0.05, ** *p* < 0.01, *** *p* < 0.001 vs. the VEGF control. Each value represents the mean ± SD from three independent experiments.

The phosphorylation of VEGFR2 and its downstream protein kinases mediates VEGF-induced angiogenesis [16]. Thus, we examined the effect of compound **9** on VEGFR2-mediated signaling in HUVECs. As shown in Figure 4A, compound **9** clearly reduced the VEGF-stimulated phosphorylation of VEGFR2 and its downstream effectors, including STAT3, AKT, and ERK1/2. In addition, the total protein level of VEGFR2 was decreased after treatment with compound **9**, whereas those of STAT3, AKT, and ERK1/2 were not significantly affected.

Matrix metalloproteinases (MMPs) participate in the degradation of the vascular basement membrane, which is required for the invasive migration of ECs [17]. As such, we assessed the effect of compound **9** on the expression of MMP-2 and MMP-9 stimulated by VEGF. As shown in Figure 4B, compound **9** markedly reduced the expression levels of MMP-2 and MMP-9 in HUVECs. Taken together, these results suggest that compound **9** may inhibit the VEGF-induced angiogenesis of HUVECs by downregulating the VEGFR2-mediated downstream signaling pathways and MMP-2/MMP-9 expression.

### 3.4. Effect of Compound ***9*** on Tumor Cell-Induced Angiogenesis in Vitro

**Figure 5:**
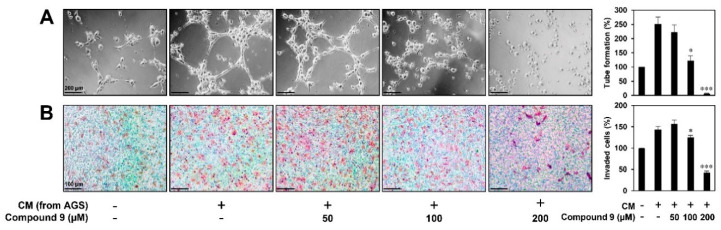
The inhibitory activity of compound **9** on tumor cell-induced angiogenesis. AGS gastric cancer cell-induced angiogenesis was assessed using (**A**) a conditioned medium from tumor cells for in vitro tube formation assay and (**B**) an in vitro co-culture system based on the chemoinvasion assay. (**A**) The basal level of the tube formation of HUVECs treated with non-conditioned medium without AGS cells was normalized to 100%. * *p* < 0.05, *** *p* < 0.001 vs. the conditioned medium from untreated AGS cells. (**B**) The basal level of the invasiveness of HUVECs that were incubated in serum-free medium without AGS cells was normalized to 100%. * *p* < 0.05, *** *p* < 0.001 vs. the control with untreated AGS cells. Each value represents the mean ± SD from three independent experiments.

Our previous study demonstrated that compound **9** exerts effective anticancer activity against AGS gastric cancer cells by inducing G2/M cell cycle arrest, apoptosis, and autophagy [15]. To further evaluate whether compound **9** affects tumor cell-induced angiogenesis, we investigated the effect of compound **9** on the invasion and tube formation of HUVECs stimulated by AGS cells. As shown in Figure 5A, HUVEC tube formation was significantly induced by culture in conditioned medium (CM) from AGS cells compared to control (medium only). However, CM from AGS cells treated with compound **9** significantly inhibited the stimulated tube formation of HUVECs. In addition, the invasion of HUVECs co-cultured with AGS cells was increased compared to that of HUVECs alone, whereas the treatment of AGS cells with compound **9** prevented the increased invasion of HUVECs (Figure 5B). These data indicate that compound **9** inhibits the in vitro angiogenesis induced by AGS gastric cancer cells.

### 3.5. Effect of Compound ***9*** on HIF-1α and VEGF Expression in Tumor Cells

**Figure 6:**
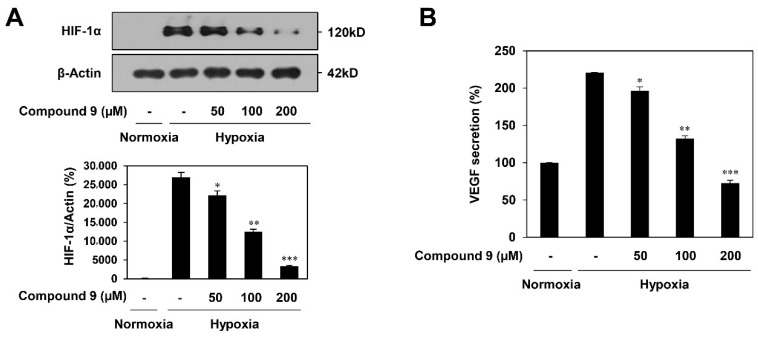
The HIF-1α inhibitory effect of compound **9**. (**A**,**B**) AGS gastric cancer cells were pretreated with compound **9** (50–200 μM) for 1 h and then exposed to 1% O_2_ for 11 h. (**A**) The effect of compound **9** on HIF-1α protein accumulation. Protein levels were detected by Western blot analysis and further quantified by densitometry. The level of β-actin was used as an internal control. (**B**) The effect of compound **9** on VEGF expression. The concentration of VEGF protein in the supernatant was determined by a VEGF ELISA. * *p* < 0.05, ** *p* < 0.01, *** *p* < 0.001 vs. the hypoxic control. Each value represents the mean ± SD from three independent experiments.

Hypoxia-inducible factor-1α (HIF-1α) plays a critical role in promoting tumor angiogenesis by activating the transcription of major proangiogenic factors, including VEGF [18]. To confirm the role of HIF-1α in mediating the suppressive effect of compound **9** on tumor cell-induced angiogenesis, we investigated the effect of compound **9** on HIF-1α expression in AGS gastric cancer cells. As shown in Figure 6A, compound **9** dose-dependently decreased the accumulation of HIF-1α protein induced by hypoxia in AGS cells. Next, we examined the effect of compound **9** on VEGF expression in AGS cells. Compound **9** inhibited VEGF secretion in AGS cells stimulated by hypoxia in a dose-dependent manner (Figure 6B). Therefore, the inhibitory activity of compound **9** on the AGS cell-induced angiogenesis may be associated with the downregulation of HIF-1α and VEGF expression.

## 4. Discussion

Tumor angiogenesis is a complicated process in which new blood vessels are formed in response to the interplay between tumor cells and ECs [19,20]. Tumor cells induce angiogenesis by secreting proangiogenic factors, such as VEGF [21]. Notably, VEGF binds to VEGFR2 on ECs with a high affinity, subsequently activating several key angiogenic signaling pathways, including STAT3, AKT, and ERK1/2, which stimulate EC growth, migration, and differentiation [22,23,24,25]. Therefore, strategies that inhibit VEGF-induced angiogenesis by targeting both ECs and tumor cells can be used to effectively block tumor angiogenesis. To the best of our knowledge, the present study is the first to demonstrate that 23-demethyl 8,13-deoxynargenicin, termed compound **9**, exhibits potential antiangiogenic activity through its inhibitory effects on VEGF/VEGFR2 signaling in ECs and the HIF-1α/VEGF pathway in tumor cells.

Nargenicin A1 has been previously found to act as an antibacterial macrolide against various Gram-positive bacteria isolated from *Nocardia* species, inhibiting DnaE involved in bacterial DNA replication [12,15,26]. In addition, nargenicin A1 has shown several other activities, including the activation of acute myeloid leukemia (AML) cell differentiation, anti-inflammation, and protection against oxidative stress [27,28,29]. Due to its valuable biological activities, various technical approaches to elucidate and modify its biosynthetic pathway have been employed to improve its production and generate novel derivatives [13,14]. In a recent study, we isolated a novel nargenicin A1 analog, termed compound **9**, in an attempt to characterize the key biosynthetic genes involved in post-polyketide synthase (PKS) tailoring of nargenicin A1 in *Nocardia* sp. CS682 [15]. Although compound **9** did not show any effective antibacterial activity as observed nargenicin A1, it exhibited potential anticancer properties, unlike the parent compound. Among the tested cancer cell lines, compound **9** most sensitively suppressed the growth of AGS gastric cancer cells without cytotoxic effects against normal cell lines. Notably, compound **9** inhibited the growth of AGS cells by inducing G2/M cell cycle arrest, ROS- and caspase-mediated apoptosis, and autophagy via the downregulation of the PI3K/AKT/mTOR pathway [15]. Furthermore, while compound **9** significantly decreased the migration and invasion of AGS cells, nargenicin A1 did not. The anti-metastatic effect of compound **9** was associated with the inhibition of MMP-2 and MMP-9, two zinc-dependent endopeptidases associated with tumor invasion and metastasis, as well as the induction of angiogenesis [15]. Although our previous study identified the anticancer activity of compound **9**, its antiangiogenic properties and underlying molecular mechanisms were not investigated.

In the present study, compound **9** was found to effectively inhibit the VEGF-stimulated angiogenic phenotypes of HUVECs, including their proliferation, invasion, capillary tube formation, and adhesion. In addition, compound **9** significantly reduced the neovascularization of the chick embryo CAM model without exhibiting toxicity against pre-existing vessels. As demonstrated by both the in vitro and in vivo results, compound **9** exhibits promising antiangiogenic activity. To elucidate the antiangiogenic mechanisms of compound **9**, we investigated its effect on endothelial VEGF/VEGFR2 signaling. As a result, compound **9** was found to inhibit the VEGF-stimulated phosphorylation of VEGFR2 and its downstream effectors, namely STAT3, AKT, and ERK1/2, in HUVECs. However, compound **9** did not significantly affect the total protein levels of the downstream effectors, but markedly reduced that of VEGFR2, implying that compound **9** may obstruct the dimerization of VEGFR2. MMPs have been previously found to degrade the vascular basement membrane to aid endothelial sprouting during angiogenesis [17]. In the present study, compound **9** markedly suppressed the expression of MMP-2 and MMP-9 stimulated by VEGF in HUVECs, indicating that it reduced MMP expression in both ECs and tumor cells.

Tumor cells express a variety of proangiogenic factors, including VEGF, through HIF-1, a heterodimeric transcription factor composed of two subunits, HIF-1α and HIF-1β [30,31,32]. HIF-1β is a constitutive nuclear protein, while HIF-1α is strongly induced in response to hypoxia, growth factor stimulation, and the activation of oncogenes. Therefore, HIF-1α is considered a crucial target for suppressing tumor angiogenesis. In the present study, compound **9** was found to effectively inhibit in vitro AGS tumor cell-induced angiogenesis. Moreover, compound **9** significantly decreased the expression of HIF-1α and VEGF induced by hypoxia in AGS cells. Taken together, our results suggest that compound **9** exerts an antiangiogenic activity via the dual downregulation of VEGF/VEGFR2-mediated signaling in ECs and HIF-1α/VEGF expression in tumor cells.

In conclusion, this study reveals for the first time the novel bioactivity and action mechanism of compound **9** and demonstrates its potential as an angiogenesis inhibitor. Further studies will be needed to identify the primary cellular target of compound **9** to improve our understanding of the molecular mechanisms responsible for its anticancer and antiangiogenic activities.
